# The impact of maternal health on child’s health outcomes during the first five years of child’s life in countries with health systems similar to Australia: A systematic review

**DOI:** 10.1371/journal.pone.0295295

**Published:** 2024-03-08

**Authors:** Shalika Bohingamu Mudiyanselage, Sithara Wanni Arachchige Dona, Mary Rose Angeles, Ishani Majmudar, Miriam Marembo, Eng Joo Tan, Anna Price, Jennifer J. Watts, Lisa Gold, Julie Abimanyi-Ochom

**Affiliations:** 1 School of Health and Social Development and Deakin Health Economics, Institute for Health Transformation, Deakin University, Geelong, Australia; 2 Department of Econometrics and Business Statistics, Monash University, Clayton, Victoria, Australia; 3 Department of Education, Victoria, Melbourne, Australia; 4 Monash University Health Economics Group (MUHEG), School of Public Health and Preventive Medicine, Monash University, Melbourne, Victoria, Australia; 5 Centre for Community Child Health, The Royal Children’s Hospital, Parkville, Victoria, Australia; 6 Population Health, Murdoch Children’s Research Institute, Parkville, Victoria, Australia; 7 Department of Paediatrics, University of Melbourne, Parkville, Victoria, Australia; Group for Technical Assistance / Asian College for Advance Studies, Purbanchal University, NEPAL

## Abstract

**Introduction:**

The first five years of life is an important developmental period that establishes the foundation for future health and well-being. Mothers play a primary role in providing emotional and physical nourishment during early childhood. This systematic review aims to explore the association between maternal health and child health in the first five years of the child’s life.

**Materials and methods:**

As primary aims, we systematically synthesised published evidence relating to the first five years of life for associations between maternal health exposures (mental, physical and Health-Related Quality of Life (HRQoL) and child health outcomes (physical health, mental health, HRQoL and Health Service Use (HSU) /cost). As a secondary aim, we explored how the above associations vary between disadvantaged and non-disadvantaged populations. The search was limited to studies that published and collected data from 2010 to 2022. The systematic review was specific to countries with similar health systems to Australia. The search was conducted in MEDLINE, CINAHL, APA PsycINFO, GLOBAL HEALTH, and EMBASE databases. The quality of the included studies was assessed by The Effective Public Health Practice Project (EPHPP) tool.

**Results:**

Thirteen articles were included in the final synthesis from the identified 9439 articles in the primary search. Six (46%) explored the association between maternal mental health and child’s physical health, two (15%) explored maternal and child’s physical health, one (8%) explored maternal and child’s mental health, one (8%) explored maternal physical health and child’s HRQoL, and three (23%) explored maternal mental health and child’s HSU. We found an association between maternal health and child health (physical and mental) and HSU outcomes but no association between maternal health and child’s overall HRQoL. The results for disadvantaged communities did not show any difference from the general population.

**Discussion and conclusion:**

Our review findings show that maternal health influences the child’s health in the first five years. However, the current evidence is limited, and the findings were primarily related to a specific maternal or child’s health condition. There was no evidence of associations of child health outcomes in healthy mothers. There is an extensive research gap investigating maternal health exposures and child outcomes in quality of life and overall health.

## Introduction

The first five years of life is a critical developmental period that establishes the trajectories for ongoing health and opportunities throughout life [1, 2]. Adverse social, health, and economic circumstances in early childhood may increase the risk of adverse consequences in life ahead [3, 4]. During the early developmental phase, parents and the living environment play a primary role in providing emotional and physical nourishment by protecting against stress and encouraging emotional and cognitive mechanisms [5–7].

As the primary carer, a mothers’ health and Health-related Quality of Life (HRQoL) during pregnancy and postpartum contribute to the child’s health and health-related outcomes, particularly in children’s early childhood [8]. Maternal illnesses and poor mental health can lead to poor health outcomes in both mother and child [9]. Previous studies have identified the factors influencing maternal physical and mental well-being, such as coexisting medical conditions (like diabetes and hypertension), ongoing stress, maternal characteristics (age, marital status, employment, education), the nature of the pregnancy, the nature of the birth, families’ living conditions and socioeconomic status [10, 11]. Recent Australian data highlights an increase in maternal morbidity in the last decade. In 2019, 15% of expectant mothers had gestational diabetes (5.6% increase from 2015) [12, 13], and in 2020, 3.4% of expectant mothers had gestational hypertension [14].

Current evidence has already shown the importance of early childhood health and well-being [15, 16]. Poor physical health increases the risk of deteriorating HRQoL, physical, social, and emotional consequences in childhood, adolescence, and adulthood. Early childhood health issues such as low birth weight, chronic health issues, or stress predict an increased risk of physical and mental health concerns later in life [17]. Persistent low quality of life (poor fundamental, social, cultural, and emotional well-being) can be a leading cause of poor mental health following frequent low-stress levels throughout life [18, 19].

HRQoL is a measure of the quality of life of an individual in the context of impairments, functional state and the influence of ill health [20]. A study (2018) that investigated child HRQoL has shown that maternal depression negatively affects a child’s quality of life (change in HRQoL -0.191, P = 0.05) [21]. However, another similar study, which investigated children aged 5–12 years, highlighted that the evidence was limited for associations between maternal HRQoL and child outcomes, especially regarding early childhood (at 5 years) quality of life [22].

Access to health services is a leading environmental factor in child development [23]. Health services access and children’s health have a bi-directional relationship influencing each other [23]. For a better early childhood, both mother and child require combination support from numerous health professionals during the pregnancy and the first few years after birth [24–26]. Access to health services involves a combination of patient needs and the health services meeting the patient’s needs (availability, cost, distance) and demand (disease burden, knowledge, self-care practices) [27–29]. However, health and environmental adversities can undermine a woman’s capacity to parent and care for themselves. This can include having the resources and knowledge to seek and access professional health care. Access to health services is a major item in social determinants of health [30], and access to quality health services influences the health equity of mothers and their children [31].

Early childhood health service visits help to identify and address childhood adversity and inequity in advance [32]. Studies have found mixed findings in the association between mother’s health and children’s Health Service Use (HSU). Some have found maternal health conditions were associated with increased HSU in children [26], whereas others have found vague associations or no association [33]. Current evidence mainly focuses on maternal mental health and children’s HSU, and findings highlight that mothers tend to use more health services for their children when mother’s mental health is poor [34, 35].

Mothers and children from disadvantaged economic, social, and psychological backgrounds face more significant health burdens than those from non-disadvantaged communities as disadvantaged mothers are prone to have higher financial burdens, rates of smoking, alcohol use, young pregnancies, live in remote areas and other poor living situations [36]. Like their parents, children raised in a disadvantaged community tend to have increased risks of poor health, low educational attainment and begin their adult life as disadvantaged [37, 38].

Given the importance of early childhood health and maternal health during the first 5 years, with limited and vague findings in the current evidence for associations in maternal and child health, quality of life and HSU/costs for children under five and their mothers, this systematic review explores the association between maternal and child health in the first five years of the child’s life. The aims of this review are to study one-way associations (mother to child) between three interrelated maternal and child outcomes: 1. health (physical and mental), 2. HRQoL and 3. health service use/cost. As a secondary aim, we explored the association variations between disadvantaged and non-disadvantaged populations. This review is only interested in the one-way association relating to how the mother’s health impacts on child’s health, not vice versa (child’s health impacts on mother’s health). Health outcomes investigated in this study include physical health, mental health, HRQoL and health service use/cost.

## Materials and methods

The systematic review was conducted according to the Preferred Reporting Items for Systematic Reviews and Meta-Analyses (PRISMA) guidelines [39]. The protocol was registered in the International Prospective Register of Systematic Reviews (PROSPERO *CRD42021277247)* [40]. Ethics approval was not required as no primary data were collected.

### Primary aim

**Fig 1:**
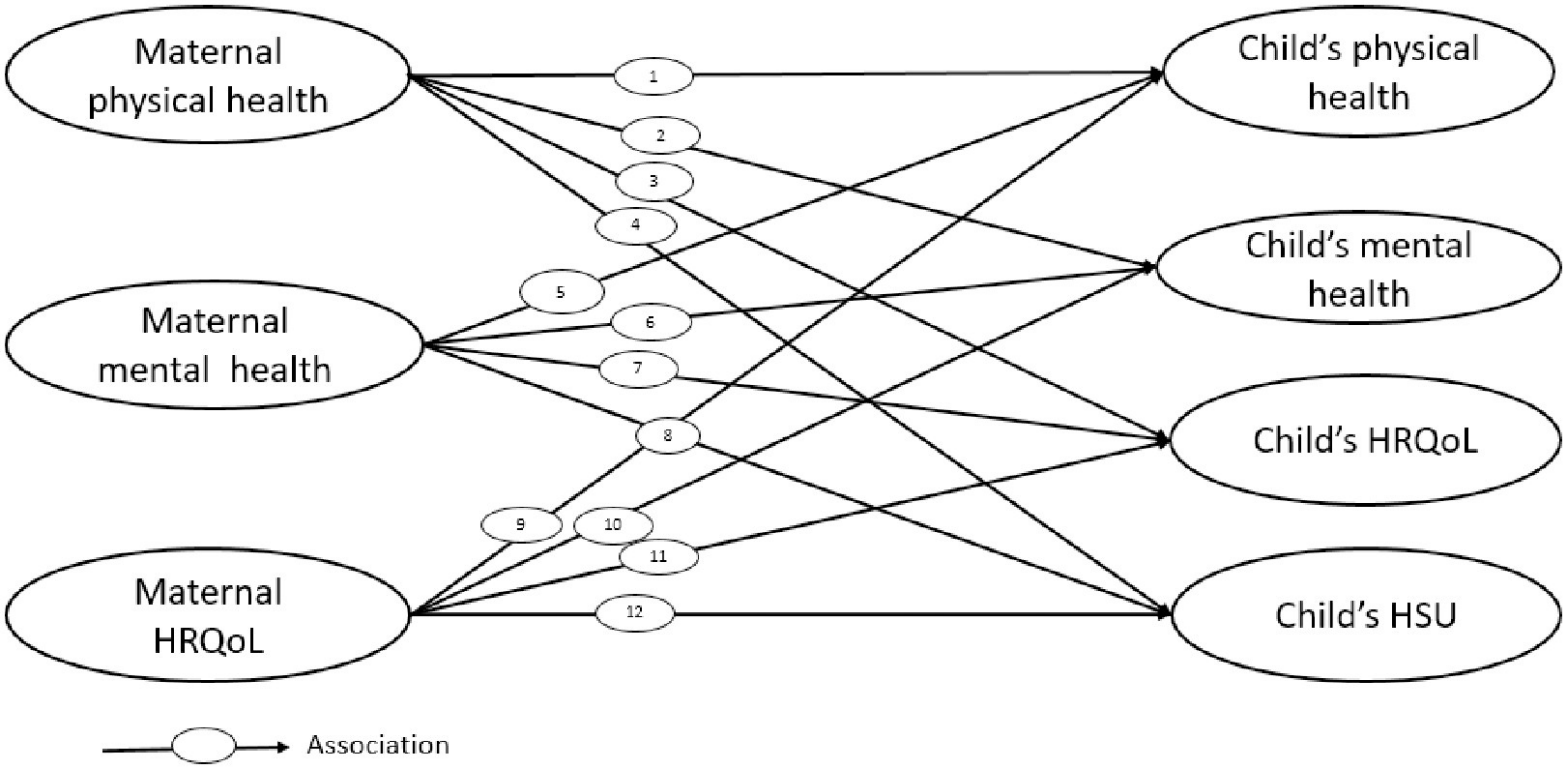
Possible associations between maternal and child health outcome. Study eligible health conditions that can affect the body’s normal functioning, considered as physical health exposures and outcomes and study eligible health conditions that affect the psychological and emotional well-being of mother or child, considered as mental health exposures and outcomes. **HRQoL**; Health-Related Quality of Life, **HSU**; Health Service Use.

To systematically synthesise published evidence relating to the first five years of life for associations between (Fig 1),

1. Maternal physical health and child health outcomes (physical health, mental health, HRQoL and HSU/cost),
2. Maternal mental health and child health outcomes (physical health, mental health, HRQoL and HSU/cost),
3. Maternal HRQoL and child health outcomes (physical health, mental health, HRQoL and HSU/cost).

### Secondary aim

1. How do the above associations vary between disadvantaged and non-disadvantaged populations?

#### Study selection

A preliminary search was conducted to identify studies examining the maternal health impact on child health outcomes as specified above. To capture recent data on the associations between maternal and child health outcomes, the systematic search was limited to studies published from 2010 to 2022 and with data collected from 2010 to 2022. The data collection period was captured in the full-text review and studies were included if the data collection period crossed the year 2010. The systematic review was specific to countries with similar health systems (in terms of the public health system, practice environment and registration indicators) to Australia (Medical Council of New Zealand) [41]. Therefore, studies were included if they were conducted in Australia, New Zealand, the United Kingdom, the United States of America, Austria, Belgium, Canada, the Czech Republic, Denmark, Finland, France, Germany, Greece, Iceland, Israel, Italy, the Netherlands, Norway, Portugal, the Republic of Ireland, Singapore, Spain, Sweden or Switzerland. The search was not limited to the English language. We expected that non-English abstracts would allow us to understand the current research on this topic, but we did not intend to include full articles of non-English publications due to limited translation resources.

The initial search for existing reviews related to maternal and child health was conducted in PROSPERO and Cochrane Controlled Trial Register, Google Scholar and EBSCO databases. The search strategy was developed by the primary reviewer (SBM) in consultation with the expert team specialised in the subject area, the review team, and the librarian. We found two systematic reviews looking at maternal health impact on children specific to prenatal infection (Simanek, 2015) [42] and anxiety (Rees, 2019) [43], both included publications/data before 2010. Simanek’s review [42] concluded that, in general, the association of maternal influenza infection during pregnancy on the child’s mental health was conflicting according to the current evidence. Further research is needed to clarify whether maternal infection with influenza and other infections increases the risk for mood disorders in children. The review by Rees [43] noted that both maternal prenatal and postnatal anxiety have a small adverse effect on child emotional outcomes overall. We did not identify any systematic review exploring associations in maternal and child health considering overall physical health, mental health, HRQoL or health service use/ cost.

The main search was conducted in MEDLINE (via EBSCO database), CINAHL (via EBSCO database), APA PsycINFO (via EBSCO database), GLOBAL HEALTH (via EBSCO database), and EMBASE electronic bibliographic databases. The search terms were based on four main concepts; 1. Maternal characteristics, 2. Child characteristics, 3. Target outcomes (health, HRQoL and HSU), and 4. Study country. Details of the search strategy are included in the S1 Table.

#### Inclusion criteria

Quantitative research studies that investigated the association between maternal and child health (five years old and under) were included. Studies conducted in countries with similar health systems to Australia, as previously defined, were included. Finally, we included maternal exposures and child health outcomes (physical health, mental health and HRQoL) if they were measured using a general measure, rating, scale, questionnaire, checklist or tool.

#### Exclusion criteria

Studies were excluded if they had limited information relating to research outcomes (protocols and conference abstracts) or investigated the child health impact on maternal health outcomes. Studies were excluded if they were (1) about mortality, cancer, HIV or a traumatic event; (2) related to lifestyle factors or behaviours (smoking, alcohol, diet, sleep); (3) related to dental health interventions; (4) in specific populations, such as disability or domestic violence, and (5) about nutrition, breastfeeding, inter-utero health, neonatal health, congenital abnormalities, immunisation or early childhood developmental interventions.

#### Study selection and data extraction

The primary reviewer (SBM) imported data from the main search to an Endnote library. After duplicate removal, data were uploaded to the Covidence systematic review and management website [44] for the title and abstract review. In Covidence, an initial screening (n = 100 studies) was conducted to test inclusion/exclusion criteria and inter-reviewer agreement. After establishing a satisfactory agreement on Covidence, two reviewers independently screened the titles and abstracts of articles. The review team consisted of six reviewers in three groups (SBM, SWD, EJT, MRS, MM, IM). The full text of all articles after the title and abstract screening were then retrieved and reviewed by two reviewers. Any discrepancies were discussed extensively within the review team, and decisions were made by consensus.

#### Quality assessment

To assess the quality of the included studies, the Effective Public Health Practice Project (EPHPP) tool was used as it covers a range of quantitative study types [45]. The tool has six components: selection bias, study design, confounders, blinding, data collection, and withdrawals and drop-outs. Each component scores as strong, moderate or weak, and a global rating score was produced by combining all six components. The EPHPP tool has content and construct validity [46]. All selected studies were included in the review regardless of their quality, and the quality rating was presented.

#### Data synthesis

We narratively synthesised the associations of physical health, mental health, HRQoL and health service use/cost exposures and outcomes.

## Results

**Fig 2:**
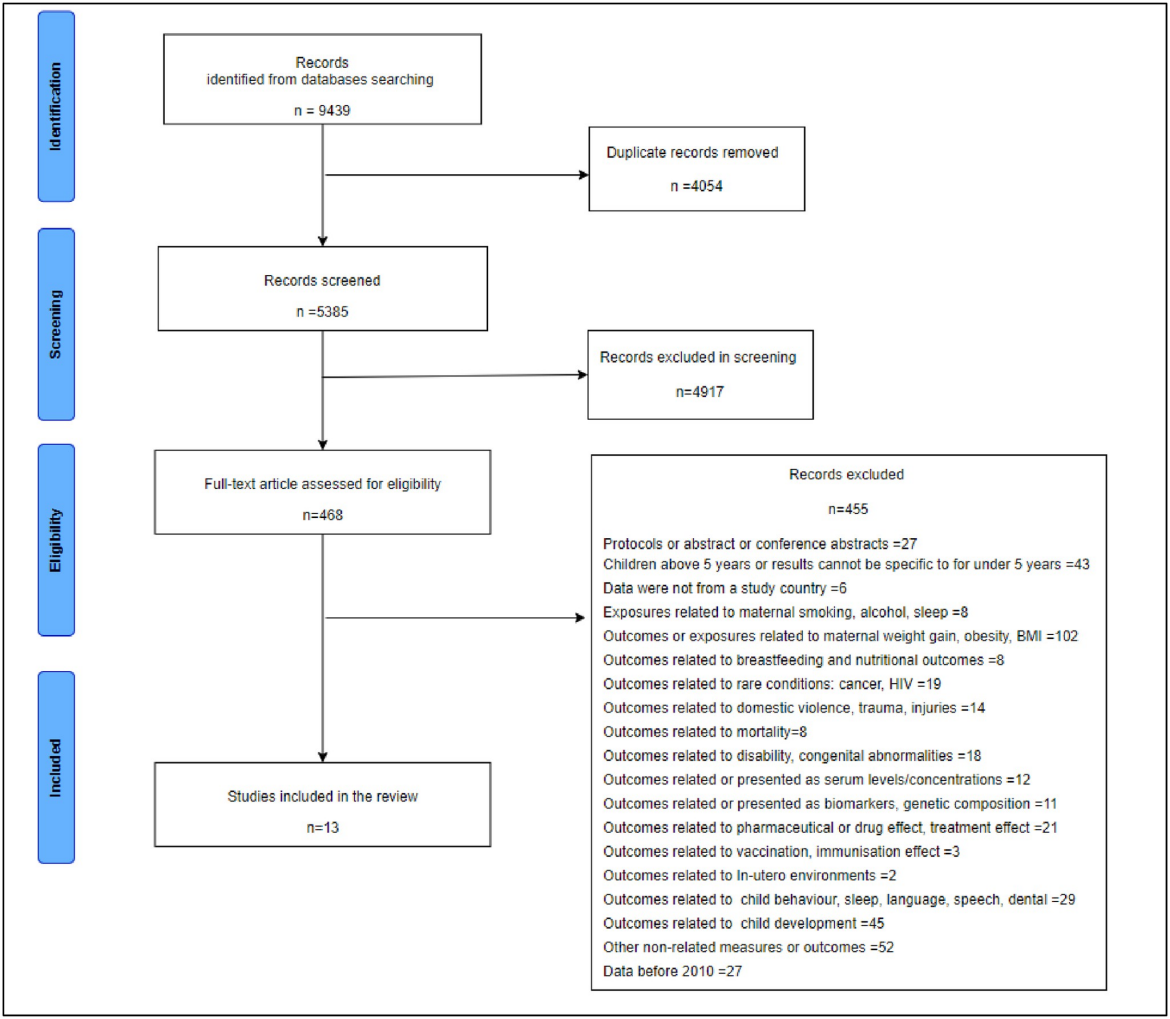
PRISMA diagram.

The search strategy identified 9439 articles, from which 13 articles were included in the final synthesis (Fig 2). From the included articles, six (46%) explored the association between maternal mental health and child’s physical health [47–51], two (15%) explored maternal and child’s physical health [9, 52], one (8%) explored maternal and child’s mental health [53], one (8%) explored maternal physical health and child’s HRQoL [54], and three articles (23%) explored maternal mental health and child’s health service use [55–57].

### Study characteristics

The study characteristics are presented in Table 1. Studies have generally investigated an association between particular health conditions (disease-specific) or the overall health of mothers and children. All articles included cohort studies that explored the data from primary studies [48–51, 53, 58], clinic/ hospital databases [47, 52, 54, 55, 57, 59], national surveys or registries [9, 56]. The data collection periods ranged from 2000 to 2019, and study participants were from Australia [9], the UK [55], the USA [49, 57], Canada [47, 52, 59], Germany [48], Denmark [56], the Netherlands [54], Finland [50, 53] and Italy [51]. One study (8%) [49] comprised a disadvantaged population, and three studies (23%) [47, 56, 59] considered socioeconomic disadvantage as a factor in study population characteristics.

**Table 1:** Study characteristics.

	Study	Target population	Data collection source/dataset and period	Main maternal exposure	Main child’s outcome	Sample size (mother and child pairs)
Maternal mental health and child’s physical health						
1	Auger N et al., 2021 Canada	Infants born in study hospitals	The Hospital Clientele Registry: 2006–2016	Mental health disorders	Infections	1. 832,290
2	Braig S et al., 2017 Germany	Mothers staying in the study hospital after delivery	The Ulm SPATZ Health Birth Cohort: 2012–2013	Chronic stress, depression and anxiety	Atopic Dermatitis (AD)	2. Participants with AD symptoms = 787
						3. Total participants = 934
3	Bush N et al., 2021 USA	Pregnant women from low-income families with 25-40kg/m^2^ BMI	The Stress, Eating, and Early Development (SEED) study: 2011–2014	Stressful life events	Infections illness and non-infection illness	109
4	Korhonen LS et al., 2019 Finland	Pregnant women performed pregnancy ultrasound scans, and were delivered at study sites	The FinnBrain Birth Cohort Study: 2011–15	Psychological distress	Recurrent Respiratory Infections (RRI)	1. Participants with RRI = 204
						2. Participants without RRI = 1,014
5	Le-Nguyen A et al., 2021 Canada	Mothers diagnosed with mental disorders before or during pregnancy	The Hospital Clientele Registry: 2006–2019	Mental health disorders	Pathological abdominal conditions	1. Participants with mental disorders = 51,371
						2. Participants without mental disorders = 1,029,147
6	Rusconi F et al., 2019 Italy	Pregnant women in the study sites	The Piccolipia Birth Cohort: 2011–2015	Mental health	Infections and wheezing	2,314
**Maternal and child’s physical health**						
1	Ahmad K et al., 2021 Australia	Infants born in 2004 and their mothers	Longitudinal Survey of Australian Children: 2004–17	General and mental health	General health and medical conditions	5,019
2	Belkaibech S et al., 2020 Canada	Infants hospitalised for Kawasaki Disease	The Hospital Clientele Registry: 2006–18	Autoimmune disorders	Kawasaki Disease (KD)	1. Participants with autoimmune = 13,239
						2. Participants without autoimmune = 778,869
**Maternal and child’s mental health**						
1	Lahti M et al., 2017 Finland	Pregnant women performed their first pregnancy ultrasound scans at study sites	The Prediction And Prevention Of Preeclampsia And Intrauterine Growth Restriction (PREDO) Study: 2011–12	Depressive symptoms	Psychiatric problems	2,296
**Maternal physical health and child’s quality of life**						
1	Giessen JV et al., 2019 The Netherlands	Pregnant women visited an Inflammatory Bowel Disease (IBD)pregnancy outpatient clinic	IBD Outpatient Clinic Dataset: 2013–16	Inflammatory Bowel Disease (IBD)	Health-related quality of life	1. Participants with IBD = 182
						2. Participants without IBD = 70
**Maternal mental health and child’s health service use**						
1	Hope H et al., 2021 UK	Children aged 0–17 years who used health services between 2007–17	Clinical Practice Research Datalink (CPRD GOLD): 2007–2017	Mental health Issues (MHI)	Healthcare resource use and cost	1. Participants with MHI = 112,741
						2. Participants without MHI = 376,5141
2	Lyngsøe BK et al., 2019 Denmark	Infants born from 2000 to 2013 and their mothers	Danish National Patient Register: 2000 to 2013	Depression	Primary healthcare use	1. Participants with depression = 78,748
			Danish Civil Registration System: 2000 to 2013			2. Participants without depression = 790,392
3	Simas TAM et al., 2019 USA	Women aged 15–50 years with at least one medical claim for delivery between 2010–15	Watson Health Market Scan Database: 2010–16	Postpartum depression (PPD)	Healthcare resource use and cost	1. Participants with PPD = 33,314
						2. Participants without PPD = 102,364

### Maternal exposures

As maternal exposures, studies explored overall general health (participant’s overall physical, mental, and social aspects of health) [9], autoimmune diseases [52] and Inflammatory Bowel Disease [54] as physical health exposures (23%). For mental health exposures (77%), studies examined mother’s overall mental health [9, 51], mental health disorders [47, 55, 59], depression [56, 57], psychological distress [50] and stress [48, 49] against child outcomes, Table 1.

### Child outcomes

Studies investigated child physical health (62%), mental health (8%), HRQoL (8%) and health service use care/cost (23%) associations with maternal exposures. Child physical health outcomes studied included atopic dermatitis [48], infections [47, 49–51], general health [9], abdominal diseases [59], Kawasaki disease [52] and wheezing [51] Lahti (2017) investigated psychiatric problems in children under five years old as mental health outcomes [53]. Giessen (2019) explored children’s HRQoL using the TNO-AZL Preschool Children Quality of Life (TAPQOL) questionnaire [54]. Simas (2019) and Hope (2021) investigated the HSU and HSU cost outcomes in children under 5 years old [55, 57].

*1*. *Association between maternal mental and child’s physical health*. Six studies investigated the associations between maternal mental health and child physical health. Studies have focused on common perinatal mental health disorders in women such as stress, depression, and anxiety. As maternal mental health associated child outcomes, four studies have looked at infectious diseases in children [47, 49–51]. All studies found that poor maternal mental health was associated with specific infectious diseases in their children up to the age of 5 years [47, 49–51]. Another two studies investigated the associations between the mother’s mental health and pathological abdominal disorders in children (Risk Ratio (RR) 1.26 (95% CI 1.09–1.46) [59] and atopic dermatitis (coefficient in Quarter (Q)1 1.3 (0.8;1.9), Q2 1.4 (0.9;2.1) Q3 1.5 (1.0;2.3), P = 0.05) [48]. Both studies found that poor maternal mental health was associated with poor physical health outcomes in their children, Table 2 and S3 Table.

**Table 2:** Maternal exposure and child outcomes relationship.

Study		Maternal		Child		Conclusion*
		Exposure and timeline	Assessment tools	Outcome and timeline	Assessment tools	
**Maternal mental health and child’s physical health**						
1	Auger N et al., 2021 Canada	**Mental health disorders** Depression, bipolar disorder, stress- and anxiety-related disorders, schizophrenia and delusional disorder, personality disorder and drug- or alcohol-related substance use disorders When **Before or during pregnancy**	1. Hospital diagnosis at admission 2. Parent-reported questionnaire 3. Outpatient clinic diagnosis	**Infections** acute upper respiratory infections, influenza, tonsillitis, bronchitis, bronchiolitis and pneumonia, infectious enteritis, appendicitis, otitis media, meningitis, encephalitis, urinary tract, myocarditis, endocarditis and pericarditis, septic arthritis, osteomyelitis, skin infections, septicemia, measles, mumps, rubella, varicella, rotavirus, poliomyelitis, hepatitis A, hepatitis B, diphtheria, tetanus, whooping cough, Haemophilus influenzae, Streptococcus pneumoniae and meningococcal infection. When **Birth to 13 years**	1. Hospital diagnosis at admission	Maternal mental health is associated with infections in their children
2	Braig S et al., 2017 Germany	**Chronic stress, depression and anxiety** when Following delivery	1. The Pregnancy-Related Anxiety Questionnaire (PRAQ-R) 2. Diagnosis in electronic hospital charts 3. The screening scale of the Trier Inventory of Chronic Stress (SSCS-TICS) 4. The Hospital Anxiety and Depression Scale(HADS)	**Atopic Dermatitis (AD)** when **at 6 months, 1 year and 2 year**	1. AD symptoms 2. Parent-reported diagnosis 3. Paediatrician reported diagnosis	Maternal stress is associated with AD in their children
3	Bush N et al., 2021 USA	**Stressful life event** When **During pregnancy (at 17.4 ±4.2 weeks, 25.6±4.5 weeks and 6 months postpartum**	1. Interviews: Phone 2. Cohen Perceived Stress Scale (PSS)	**Infections illness and non-infection illness** when **Birth to 1**^**st**^ **year**	1. Medical record abstraction 2. Medication prescriptions 3. Surgical procedures	Maternal perceptions of stress across pregnancy were associated with infant illness. Stressful life events and postnatal stress were not associated with children’s infectious illness
4	Korhonen LS et al., 2019 Finland	**Psychological distress**: symptoms of depression and anxiety and relationship satisfaction /quality When **During pregnancy**	1. Edinburgh Postnatal Depression Scale (EPDS) 2. Symptom Checklist-90 anxiety sub-scale 3. Pregnancy-Related Anxiety Questionnaire (PRAQ-R2) 4. Revised Dyadic Adjustment Scale(RDAS)	**Recurrent Respiratory infections (RRI)** When **At 3 months, 6 months, 1 year and 2 year**	1. Parent-reported questionnaire	Maternal psychological distress is associated with RRI in their children
5	Le-Nguyen A et al., 2021 Canada	**Mental health disorders** when Before and during pregnancy	1. Diagnosis in Hospital database	**Pathological abdominal disorders** when **Birth to 1**^**st**^ **year**	1. Diagnosis in Hospital database	Maternal mental disorders are associated with hypertrophic pyloric stenosis in their children
6	Rusconi F et al., 2019 Italy	**Mental health** general non-psychotic psychiatric morbidity When **last 4 weeks of pregnancy and 12 months** **after delivery**	1. Generalised Health Questionnaire (GHQ): GHQ‐12	**Respiratory tract infection, diarrhea and wheezing** otitis, pharyngitis, bronchitis or bronchiolitis, pneumonia and viral gastroenteritis When **1 to 2 years**	1. Parent-reported questionnaire	Maternal mental health is associated with infection and wheezing in their children
	**Maternal and child’s physical health**					
1	Ahmad K et al., 2021 Australia	**General and mental health** General health status, presence of a medical/chronic condition (asthma, gestational diabetes, nausea, hypertensive disorder), and mental health status When **During pregnancy**	1. 5-point Likert scale 2. Clinical diagnosis 3. Medication prescriptions 4. Questionnaire from the Kessler K6 screening scale	**General health** General health status, presence of a medical/chronic condition, and mental health status (wheezing, bronchiolitis, asthma, eczema, food or digestive allergies, ear infections, hearing problems, vision problems, attention deficit disorder, other illnesses, and other infections) When **Birth to 1 year**	1. 5-point Likert scale 2. Clinical diagnosis 3. Medication prescriptions 4. *Physical health outcome index*	Maternal mental health and chronic condition in their children Maternal chronic conditions are associated with chronic conditions in their children
2	Belkaibech S et al., 2020 Canada	**Autoimmune disease** rheumatologic disorders, vasculitis, and traditional autoimmune diseases When **Before and during pregnancy**	1. Discharge summary after the delivery	Kawasaki disease When **Birth to 12 years**	1. Hospitalisation diagnosis	Maternal autoimmune conditions are associated with Kawasaki disease in their children
**Maternal and child’s mental health**						
1	Lahti M et al., 2017 Finland	**Depressive Symptoms** When **Before and during pregnancy**	**1.** Medical reports independently verified by a clinical jury and from the Finnish Medical Birth Register.	**Psychiatric Problems** Internalising, externalising, anxious, depressed, somatic complaints, withdrawn, sleep problems, attention problems, and aggressive behaviour, pervasive developmental, attention-deficit/hyperactivity, and oppositional defiant problems) When **1.5 to 5 years**	1. Child behaviour Checklist 2. Five DSM-oriented scales	Maternal depressive symptoms are associated with psychotic problems in their children
**Maternal physical health and child’s quality of life**						
1	Giessen JV et al., 2019 The Netherlands	**Inflammatory bowel disease (IBM)** When **Before and during pregnancy**	1. Clinical diagnosis	**Health-related quality of life** When **Birth to 5years**	1. TNO-AZL Preschool Children Quality of Life Questionnaire (TAPQOL)	No association between maternal IBD and children’s HRQoL
**Maternal mental health and child’s health service use**						
1	Hope H et al., 2021 UK	**Mental Health Issues** non-affective psychosis, affective psychosis, depression, anxiety, eating disorders, personality disorders and substance and alcohol misuse disorders When **After birth**	1. Clinical diagnosis 2. Prescriptions	**Health service use and costs** Primary and secondary healthcare When **Birth to 17 years**	1. primary care contacts 2. Prescriptions 3. hospital admissions outpatient visits 4. emergency department visit	Maternal depression is associated with children’s primary and secondary health services visits and costs
2	Lyngsøe BK et al., 2019 Denmark	**Depression** When **During pregnancy**	1. Records in Danish National Health Insurance Service Register 2. Reimbursement of antibiotics 3. Hospital admissions 4. outpatient contact	**Health service use** primary healthcare: General practice visits When **Birth to 2 years**	1. Records in Danish National Health Insurance Service Register	Maternal depression is associated with children’s primary health services visits and costs
3	Simas TAM et al., 2019 USA	**Postpartum Depression** When **12 months after birth**	1. Clinical diagnosis 2. Prescriptions	**Health service use and costs** When **Birth to 2 years**	1. Insurance claims	Maternal depression is associated with children’s health service visits and costs

*Detailed information on the statistical association measures is presented in the S3 Table.

*2*. *Association between maternal and child’s physical health*. Two studies investigated maternal and child physical health during pregnancy. Ahmad and colleagues (2021) showed that poor general health of the mother in the year after childbirth was associated with poor health in infants (Odds Ratio (OR) 3.13 (95% CI 2.16;4.52)). Furthermore, the study revealed that having any chronic condition in mothers during pregnancy significantly increased the likelihood of chronic conditions in their children during infancy (OR: 1.31 (95% CI 1.12;1.54)). Belkaibech (2020) found that when the mother suffers from autoimmune thyroiditis before or/and during pregnancy, the child’s risk of Kawasaki disease was Hazard Ratio (HR) of 10.98 (95% CI 9.42;12.81) in the first year and compared to HR of 8.11 (95% CI 7.15;9.21) in the second year. However, child’s risk of getting any other autoimmune disease (rheumatologic disorders, vasculitis, traditional autoimmune diseases, autoimmune thyroiditis) when the mother suffers from an autoimmune disease is much less during the first 2 years than getting Kawasaki disease.

*3*. *Association between maternal and child’s mental health*. One study by Lahti (2017) investigated the relationship between maternal mental health and child’s mental health (1.5–5 years) and found that mental health disorders (such as internalising, externalising, anxiety, depression, sleep problems, attention problems) were common in children whose mothers had clinically significant depression across pregnancy trimesters and during and after pregnancy (internalising problems, Coefficient 0.28 (95% CI 0.25;0.32), externalising problems 0.26 (95% CI 0.23;0.30) and total problems 0.31 (95% CI 0.27;0.35)) [53].

*4*. *Association between maternal physical health and child’s quality of life*. A study by Giessen (2019) investigated the association between mothers with Inflammatory Bowel Disease (IBD) and children’s HRQoL for those aged 1–5 years. Children’s overall HRQoL, as reported by the parents [54], did not differ between children of mothers with and without IBD (median 90 (86.5–93.3) vs. 89.1 (84.4–92.3, P = .18), suggesting no association between this maternal physical health condition (IBD) and child HRQoL [54].

*5*. *Association between maternal mental health and child’s health service use*. Three studies from Denmark, UK and USA, examined mothers’ mental health and child’s HSU during the first five years of life. The findings from these studies consistently highlighted an association between maternal depression and child’s HSU. In Denmark, Lyngsøe (2019) showed that maternal depression was associated with higher use of primary health services for their children. The association was stronger when mothers were diagnosed recently (0–6 months), resulting in their children having 16% more General Practitioner (GP) contacts than children of a mother without a diagnosis of depression (adjusted IRR = 1.16 (95% CI 1.15; 1.17)).

The USA study by Simas (2019) used medical claims to investigate HSU and cost in young children (0–2 years) of mothers with Postpartum Depression (PPD). During the 2-year follow-up, HSU (across most HSU categories; Emergency Department (ED), outpatient and physician office visits) was significantly higher among children whose mothers suffered from PPD compared to the non-PPD group (the percentage of children with a physician specialist service was 68% vs. 64%, early-intervention screening was 40% vs. 37%, and an ED visit was 48% vs. 42%, all P<0.001). Children of mothers with PPD incurred 12% higher total HSU costs in the first two years than children of mothers without PPD (mean cost US$28,527 versus U$25,478; P<0.001, in 2022 US$). Mother’s medical claims, as well as the proportion of children with ED visits, physician specialist services, and outpatient pharmacy claims, were significantly higher in the PPD exposure cohort than the non-PPD exposure cohort (all P<0.001).

A UK study by Hope (2021) examined maternal mental disorders and children’s (0–17 years) HSU (primary care, prescriptions, referrals, outpatient visits, hospitalisations, ED visits) and costs. The highest annual health service costs: for children exposed to maternal mental disorders, was £3,076 (US$4,616 in 2022) per child per year compared with £2,211 (US$3,318 in 2022) for unexposed children, and the mean cost difference was £864 (95% CI £810;918) in 2022 (US$1,297 (95% CI 1,215;1,378)) per exposed child per year.

### Disadvantaged populations

A study undertaken by Bush (2021) targeted a disadvantaged population with an income of 500% or less of the federal poverty level, racially/ethnically diverse, overweight and highly stressed [49]. The study found that prenatal maternal stress (measured Cohen Perceived Stress Scale) is associated with infant illness during the first year of life. Specifically, each one-point increase in average stress using the Cohen scale was associated with a 38% increase in the incidence of infants’ infection (IRR 1.38(95% CI 1.01;1.88), P<0.05), and a 73% increase in non-infectious illness (IRR, 1.73 95% CI 1.34;2.23), P<0.05) [49].

### Quality of the studies

Quality ratings are presented in the S2 Table. Most studies (10/13, 77%) had a moderate global rating. Two (15%) had a weak global rating, and only one (8%) study showed a strong global rating using the EPHPP assessment tool.

## Discussion

Our review findings show associations between maternal health and the child’s health in the first five years. The review found an association between maternal health and child health (physical and mental) and HSU outcomes but no evidence of the association between maternal health and child’s overall HRQoL. Results reveal that the mother’s poor general health (participants’ overall physical, mental, and social aspects of health) chronic conditions, and specific conditions such as autoimmune disease are associated with the child’s physical and mental health, whereas mothers’ poor physical health influenced poor physical and mental health in their children. However, there are research gaps in investigating overall maternal health (without any health condition) effects on a child’s health, HRQoL and HSU/cost in early childhood. In summary, our review found studies that investigated the associations between:

- maternal mental health and child’s physical health
- maternal and child’s physical health
- maternal and child’s mental health
- maternal physical health and child’s quality of life
- maternal mental health and child’s health service use

However, we did not find any studies that investigated the associations between:

- maternal physical health and child mental health
- maternal physical health and child HSU/cost
- maternal mental health and child HRQoL
- maternal HRQoL and child physical health
- maternal HRQoL and child mental health
- maternal HRQoL and child HRQoL
- maternal HRQoL and child HSU/cost

**Fig 3:**
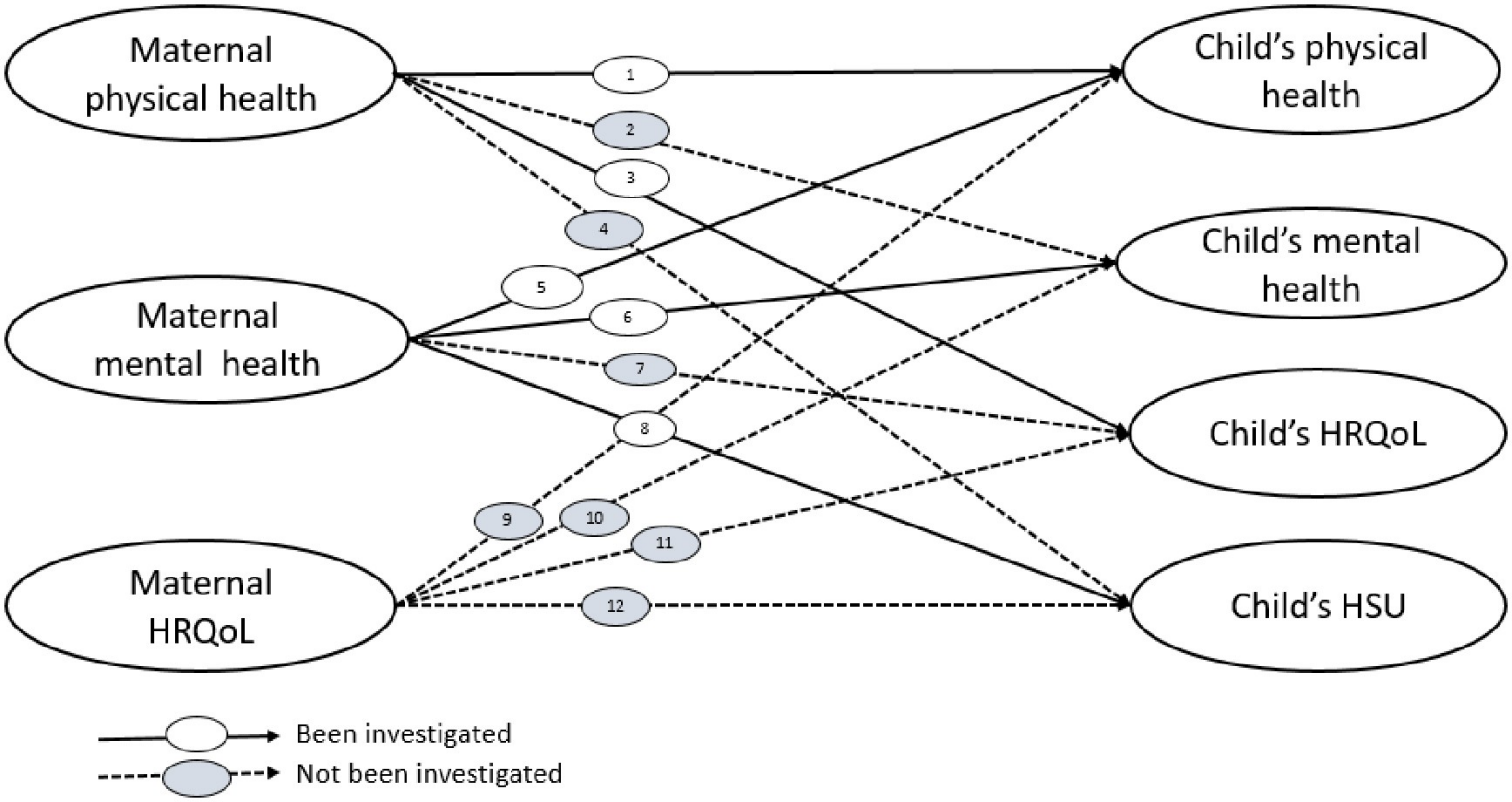
Current evidence on possible associations between maternal and child health outcomes. **HRQoL**; Health-Related Quality of Life, **HSU**; Health Service Use.

Across 12 possible associations (Fig 3) between maternal and child health outcomes, only five (42%) associations have been investigated, and the remaining seven (58%) associations have not been explored after 2010, highlighting the current evidence gap.

Further, from the above possible 12 associations, we only found evidence for one (8%) association that investigated a disadvantaged community. This highlights the need for fresh research approaches to reveal the links and factors that influence maternal and child health specific to disadvantaged communities in high-income countries. The variation in the association between maternal physical health and child outcomes is extensive in the literature, and the evidence of maternal morbidity’s impact on children requires further research attention [60]. The literature has no consistent findings relating to the associations between maternal physical health and child outcomes.

Many factors associated with poor maternal health (such as nature or the phase of the maternal health condition, stress due to the illness, family and social support circle and many other factors) could influence children’s HRQoL. Similar to maternal health, maternal HRQoL can also influence the child’s health [61]. However, based on one study captured in our review, there was no association between maternal health and child HRQoL [54]. Measuring HRQoL in children under five is complex, as results can vary on reporting person perspectives and follow-up time points. Many paediatric quality-of-life instruments are reported as caregivers-proxy [62], and some instruments can be used as self-reporting but can only be used for distinct dimensions of HRQoL for very young children [60]. As it is not possible to draw conclusions using the one available study, this highlights another significant research gap.

Current research mainly focuses on mental health impacts on mothers and children, especially in the perinatal period. Following the same pattern, in this review 89% of included studies have investigated associations between maternal mental health and/or child mental health [47–50, 53, 55–57]. The findings are consistent with the associations between maternal mental health and child outcomes. The results conclude that poor maternal mental health is associated with poor health in their children during the first five years of their life. Besides the impact of maternal mental health on a child’s life trajectory, poor maternal mental health increases health service costs and productivity loss for mothers, adding more significant personal and financial burdens on mothers, society and the child for decades [63, 64].

Our review results also reveal that mothers with depression use more health services for children, resulting in higher HSU costs. Evidence suggested that this could be because they are more likely to seek medical care for their child’s minor injuries due to stress, depression, or anxiety [65], or health professionals might be more likely to have extra contact with the children of parents with poor health and socioeconomic state [66].

Our review found only one study on socioeconomically disadvantaged populations in developed countries and did not see a different pattern of findings compared to the general population. However, it is difficult to draw conclusions from one study. This review included high-income countries where disadvantaged communities are often supported for service provision (antenatal care packages, family welfare and tax benefits, national care plans and research) [67–69]. Further research in different dimensions is needed for these communities in high-income countries to further understand the determinants of maternal and child health, HRQoL and HSU.

There are several strengths to this review. This is the first systematic review to investigate all physical, mental, HRQoL and HSU associations between mothers and children over the first five years of life. Studies in this review collected maternal exposures and child outcome data based on clinical diagnosis or standard data collection tools.

As a limitation, due to the broadness of the topic "maternal and child health" and the limited resources (time), we only included studies in the last 12 years, aiming to collect recent evidence.

## Conclusion

Our review found an association between maternal health and child health (physical and mental) and health service use outcomes but no association between maternal health and child’s overall HRQoL. However, the finding was primarily related to a specific maternal or child’s health condition. There was no evidence of associations of child health outcomes in healthy mothers. There is an extensive research gap investigating maternal health exposures and child outcomes in quality of life and overall health. Further, there is a need for comprehensive research to examine the associations between maternal and child health outcomes among disadvantaged populations in high-income countries.

## Supporting information

S1 Table Search strategy. (DOCX)

S2 Table Quality assessment results. (DOCX)

S3 Table Study association measures results. (DOCX)

S1 Checklist PRISMA 2009 checklist. (PDF)
