# Supplementary material for: The impact of maternal health on child’s health outcomes during the first five years of child’s life in countries with health systems similar to Australia: A systematic review
Source: PLoS One. 2024 Mar 8;19(3):e0295295. doi: 10.1371/journal.pone.0295295 (PMC10923423; doi:10.1371/journal.pone.0295295)
Supplement: S1 Table — (DOCX) [file pone.0295295.s001.docx]

##### S1 Table: Search strategy

| Concept 1: Maternal characteristics | Search location |
| --- | --- |
| maternal OR mother* OR pregnan* OR prenatal OR "pre natal" OR "post natal" OR postnatal OR antenatal OR "ante natal" OR gestation* | Title |
| Concept 2: Child's characteristics |  |
| baby OR infant* OR babies OR newborn* OR toddlers* OR "pre schooler*" OR Child* OR paediatric OR pediatric* OR "under 5 year*" OR offspring* | Title |
| Concept 3: outcomes |  |
| health OR illness* OR disorder* OR symptom* OR depression OR "quality of life" OR "QOL" OR "HRQOL" | Title and abstract |
| concept 4: Geographic location |  |
| Australia OR "New Zealand" OR Austria OR Belgium OR Canada OR Czech OR Denmark OR Finland OR France OR Germany OR Greece OR Iceland OR Israel OR Italy OR Norway OR Portugal OR Ireland OR Singapore OR Spain OR Sweden OR Switzerland OR Netherland OR "United Kingdom" OR England OR Britain OR British OR America OR "United States" | Title, abstract and subject heading |
