# Supplementary material for: The impact of maternal health on child’s health outcomes during the first five years of child’s life in countries with health systems similar to Australia: A systematic review
Source: PLoS One. 2024 Mar 8;19(3):e0295295. doi: 10.1371/journal.pone.0295295 (PMC10923423; doi:10.1371/journal.pone.0295295)
Supplement: S2 Table — (DOCX) [file pone.0295295.s002.docx]

##### S2 Table: Quality assessment results

|  | **Selection bias** | **Study design** | **Confounders** | **Blinding** | **Data collection methods** | **Withdrawals and dropouts** | **Global rating** |
| --- | --- | --- | --- | --- | --- | --- | --- |
| 1. Auger N et al., 2011   Canada | Moderate | Moderate | Moderate | Moderate | Strong | Strong | Moderate |
| 1. Braig S et al., 2017   Germany | Weak | Moderate | Moderate | Moderate | Strong | Moderate | Moderate |
| 1. Bush N et al., 2021   USA | Moderate | Moderate | Moderate | Moderate | Strong | Strong | Moderate |
| 1. Korhonen LS et al., 2019   Finland | Weak | Moderate | Weak | Moderate | Strong | Weak | Weak |
| 1. Le-Nguyen A et al., 2021 Canada | Moderate | Moderate | Moderate | Moderate | Strong | Strong | Moderate |
| 1. Rusconi F et al., 2019   Italy | Moderate | Moderate | Moderate | Moderate | Strong | Moderate | Moderate |
| 1. Ahmad K et al., 2021   Australia | Moderate | Moderate | Moderate | Moderate | Moderate | Moderate | Moderate |
| 1. Belkaibech S et al., 2020   Canada | Moderate | Moderate | Moderate | Moderate | Strong | Strong | Moderate |
| 1. Lahti M et al., 2017   Finland | Weak | Moderate | Moderate | Moderate | Strong | Moderate | Weak |
| 1. Giessen JV et al., 2019   The Netherlands | Moderate | Moderate | Moderate | Moderate | Strong | Moderate | Moderate |
| 1. Hope H et al., 2021   UK | Moderate | Moderate | Moderate | Strong | Strong | Moderate | Moderate |
| 1. Lyngsoe BK et al., 2019   Denmark | Strong | Moderate | Moderate | Moderate | Strong | Strong | Strong |
| 1. Simas TAM et al., 2019   USA | Moderate | Moderate | Moderate | Moderate | Strong | Strong | Moderate |
