# Supplementary material for: The impact of maternal health on child’s health outcomes during the first five years of child’s life in countries with health systems similar to Australia: A systematic review
Source: PLoS One. 2024 Mar 8;19(3):e0295295. doi: 10.1371/journal.pone.0295295 (PMC10923423; doi:10.1371/journal.pone.0295295)
Supplement: S3 Table — (DOCX) [file pone.0295295.s003.docx]

**S3 Table: Study association measures results**

**Table abbreviations; CE: coefficient, RR: rate ratio, HR: hazard ratio, IRR: incident rate ratio, OR: odds ratio, PPD: postpartum depression, MMI: maternal mental illness**

| **Study** | **Exposure (age measured)** | **Outcome(age measured)** | **Association measure results** | | | | |
| --- | --- | --- | --- | --- | --- | --- | --- |
| Braig S et al., 2017 | Chronic stress, depression and anxiety (soon after delivery) | Atopic Dermatitis (6 months, 1 year and 2 year) |  | With mental illness, CE (95% CI) | Without mental illness CE (95% CI) |  |  |
|  |  |  | Screening scale of the Trier Inventory of Chronic Stress(SSCS-TICS) | P=0.042 | P=0.05 |  |  |
|  |  |  | Q2 | CE 1.3(0.8;1.9) | CE 1.3(0.8;1.9) |  |  |
|  |  |  | Q3 | CE 1.3(0.9, 2.1) | CE 1.4(0.9;2.1) |  |  |
|  |  |  | Q4 | CE 1.5(1.0;2.3) | CE 1.5(1.0;2.3) |  |  |
|  |  |  | Pregnancy-Related Anxiety Questionnaire(PRAQ-R) | P=0.049 | P=0.102 |  |  |
|  |  |  | Q2 | CE 1.4(0.9;2.1) | CE 1.4(0.9;2.1) |  |  |
|  |  |  | Q3 | CE 1.5(1.0;2.3) | CE 1.4(0.9;2.2) |  |  |
|  |  |  | Q4 | CE 1.6(1.0;2.5) | CE 1.5(0.9;2.4) |  |  |
| Le-Nguyen A et al., 2021 | Maternal mental health disorders  (before and during pregnancy) | Pathological abdominal diseases  (birth to 1 year) |  | RR(95%CI), Unadjusted | RR(95%CI), Adjusted |  |  |
|  |  |  | Any mental disorder | RR 1.40 (1.21–1.62) | RR 1.26 (1.09–1.46) |  |  |
|  |  |  | Depression | RR 1.57 (1.21–2.04) | RR 1.41 (1.09–1.83) |  |  |
|  |  |  | Bipolar | RR 1.69 (1.03–2.76) | RR 1.53 (0.93–2.50) |  |  |
|  |  |  | Schizophrenia | RR 0.79 (0.33–1.90) | RR 0.72 (0.30–1.71) |  |  |
|  |  |  | Stress and anxiety | RR 1.39 (1.18–1.64) | RR 1.26 (1.06–1.49) |  |  |
|  |  |  | Personality disorder | RR 1.32 (0.99–1.75) | RR 1.13 (0.85–1.50) |  |  |
|  |  |  |  |  |  |  |  |
|  |  |  | Any abdominal disorder | RR 1.26 (1.09–1.46) |  |  |  |
|  |  |  | Hirschsprung disease | RR 1.15 (0.67–1.96) |  |  |  |
|  |  |  | Hypertrophic pyloric stenosis | RR 1.39 (1.16–1.68) |  |  |  |
|  |  |  | Esophageal atresia | RR 1.08 (0.64–1.83) |  |  |  |
|  |  |  | Intestinal atresia | RR 1.12 (0.81–1.55) |  |  |  |
|  |  |  | Small intestine | RR 0.83 (0.49–1.41) |  |  |  |
|  |  |  | Large intestine | RR 1.44 (0.97–2.14) |  |  |  |
|  |  |  | Biliary atresia | RR 0.66 (0.23–1.88) |  |  |  |
| Bush N et al., 2021 | Stressful life event (at 17.4 weeks gestation, 25.6 gestation weeks and 6 months postpartum ) | Infections and non-infection illness (Birth to 1 year) |  | Infectious diseases, IRR (95% CI) | Non-infectious diseases, IRR (95% CI) |  |  |
|  |  |  | Prenatal stress | IRR 1.38,(1.01;1.88),<0.05 | IRR 1.73(1.34;2.23),<0.01 |  |  |
|  |  |  | Late pregnancy | IRR 1.55,(1.18;2.03),<0.01 | IRR 1.83(1.43;2.25),<0.01 |  |  |
|  |  |  |  |  |  |  |  |
| Korhonen LS et al., 2019 | Psychological distress (34 weeks gestation) | Recurrent Respiratory infections (3 months, 6 months, 1 year and 2 year) |  | Respiratory infections at 2 year, OR (95% CI), P-value |  |  |  |
|  |  |  | Edinburgh Postnatal Depression Scale(EPDS) | OR 1.24(1.08;1.44),0.003 |  |  |  |
|  |  |  | Pregnancy-Related Anxiety Questionnaire–Revised 2(PRAQ-R2) | OR 1.28(1.11;1.47),0.001 |  |  |  |
|  |  |  | Revised Dyadic Adjustment Scale(RDAS) | OR 1.32(1.01;1.58),0.003 |  |  |  |
|  |  |  | Symptom Checklist 90 anxiety subscale(SCL-90) | OR 1.40(1.01;1.76),0.003 |  |  |  |
|  |  |  |  |  |  |  |  |
| Auger N et al., 2011 | Mental disorders (before and during pregnancy) | Infections (Birth to 5 years) |  | <1 yr, HR (95% CI) | 1–2 yrs, HR (95% CI) | 3–4 yrs, HR (95% CI) | >=5 yrs, HR (95% CI) |
|  |  |  | Any infection | HR 1.54(1.50;1.58) | HR 1.35(1.31;1.39) | HR 1.29(1.22;1.37) | HR 1.23(1.15;1.32) |
|  |  |  | Otitis media | HR 1.63(1.54;1.72) | HR 1.35(1.31;1.40) | HR 1.27(1.19;1.35) | HR 1.29(1.17;1.41) |
|  |  |  | Respiratory | HR 1.58(1.52;1.63) | HR 1.45(1.39;1.51) | HR 1.42(1.30;1.54) | HR 1.42(1.28;1.59) |
|  |  |  | Gastrointestinal | HR 1.85(1.70;2.01) | HR 1.54(1.43;1.67) | HR 1.57(1.38;1.79) | HR 1.27(1.13;1.43) |
|  |  |  | Central nervous system | HR 1.39(1.14;1.70) | HR 1.05(0.65;1.71) | HR 1.42(0.90;2.26) | HR 1.04(0.63;1.74) |
|  |  |  | Urinary tract | HR 1.44(1.33;1.55) | HR 1.64(1.43;1.88) | HR 1.44(1.11;1.87) | HR 1.57(1.21;2.04) |
|  |  |  | Carditis | HR 2.31(1.32;4.03) | – | – | HR 3.77(1.62–8.78) |
|  |  |  | Septic arthritis and osteomyelitis | HR 0.73(0.35;1.54) | HR 0.93(0.63;1.36) | HR 0.52(0.23;1.18) | HR 1.03(0.61;1.75) |
|  |  |  | Integument | HR 1.45(1.20;1.75) | HR 1.65(1.44;1.89) | HR 1.19(0.95;1.50) | HR 1.20(0.95;1.51) |
|  |  |  | Septicemia | HR 1.11(0.86;1.44) | HR 1.83(1.33;2.53) | HR 0.96(0.51;1.83) | HR 1.28(0.72;2.31) |
|  |  |  | Vaccine-preventable | HR 1.51(1.30;1.76) | HR 1.32(1.12;1.54) | HR 1.59(1.16;2.18) | HR 1.11(0.72;1.69) |
|  |  |  | Other infection | HR 1.48(1.36;1.62) | HR 1.57(1.42;1.73) | HR 1.51(1.28;1.78) | HR 1.23(1.02;1.49) |
|  |  |  |  |  |  |  |  |
| Rusconi F et al., 2019 | Mental disorders (late 4 weeks of pregnancy and after delivery) | Respiratory tract infection, diarrhea and wheezing(1-2 year) | General Health Questionnaire‐12(GHQ‐12) | High prenatal-low postnatal GHQ-12, RR(95% CI) | Low prenatal-high postnatal GHQ-12, RR(95% CI) | High prenatal- high postnatal GHQ-12, RR(95% CI) |  |
|  |  |  | Wheezing | RR 1.30(1.01;1.67) | RR 1.10(0.85;1.42) | RR 1.42(1.12;1.80) |  |
|  |  |  | Recurrent wheezing | RR 1.19(0.84;1.69) | RR 1.02(0.72;1.45) | RR 1.48(1.06;2.04) |  |
|  |  |  | Upper respiratory infections | RR 1.16(1.03;1.31) | RR 1.13(1.01;1.26) | RR 1.21(1.09;1.36) |  |
|  |  |  | Recurrent upper respiratory infections | RR 1.28(0.90;1.82) | RR 1.28(0.92;1.78) | RR 1.44(1.06;1.97) |  |
|  |  |  | Lower respiratory infections | RR 1.18(0.94;1.48) | RR 1.27(1.03;1.57) | RR 1.30(1.05;1.59) |  |
|  |  |  | Diarrhoea | RR 1.48(1.21;1.82) | RR 1.25(1.02;1.54) | RR 1.49(1.23;1.81) |  |
|  |  |  |  |  |  |  |  |
| Ahmad K et al., 2021 | General health, chronic conditions and mental health (during pregnancy) | General health (birth to 1 year) |  | Risk of having poor general health, OR (95%CI),P-value | Risk of having any of the selected medical conditions, OR (95%CI),P-value | Physical health outcome index score, OR (95%CI),P-value |  |
|  |  |  | Poor general health | OR 3.13(2.16;4.52),<0.05 | OR 1.10(0.92;1.31), >0.05 | OR-0.40(-1.00;0.21),>0.05 |  |
|  |  |  | Medical condition =>1 | OR 1.22(0.86;1.74),>0.05 | OR 1.31(1.12;1.54), <0.05 | OR 0.04(-0.49; 0.56),>0.05 |  |
|  |  |  | Stress, anxiety or depression | OR 0.71(0.44;1.13),>0.05 | OR 1.21(0.97;1.50),<0.05 | OR 0.93(-1.77;0.10),<0.05 |  |
|  |  |  | Stressful life events =>1 | OR 1.08(0.74;1.58),>0.05 | OR 0.91(0.77;1.07),>0.05 | OR-0.01(-0.55;0.53),>0.05 |  |
|  |  |  | Psychological distress | OR 1.12(0.86;1.47),>0.05 | OR 1.22(1.05;1.42),<0.05 | OR 0.01(-0.51;0.53),>0.05 |  |
|  |  |  |  |  |  |  |  |
| Belkaibech S et al., 2020 | Autoimmune disease (before and during pregnancy) | Kawasaki disease (Birth to 12 months) |  | 1 year, HR (95%CI) | 2 years, HR (95%CI) | 5 years, HR (95%CI) | All ages, HR (95%CI) |
|  |  |  | Any autoimmune disorder | HR 1.75(1.51;2.03) | HR 1.69(1.47;1.94) | HR 1.32(1.10;1.58) | HR 1.58(1.39;1.79) |
|  |  |  | Rheumatologic | HR 1.65(1.42;1.91) | HR 1.70(1.48;1.95) | HR 2.06(1.77;2.41) | HR 1.84(1.63;2.09) |
|  |  |  | Vasculitis | HR 2.21(1.86;2.62) | HR 1.87(1.61;2.16) | HR 0.57(0.42;0.79) | HR 1.50(1.32;1.71) |
|  |  |  | Traditional autoimmune disease | HR 2.57(2.19;3.00) | HR 2.20(1.91;2.52) | HR 0.73(0.56;0.96) | HR 1.81(1.59;2.04) |
|  |  |  | Autoimmune thyroiditis | HR 10.98(9.42;12.81) | HR 8.11(7.15;9.21) | HR 0.97(0.72;1.31) | HR 6.10(5.48;6.80) |
|  |  |  |  |  |  |  |  |
| Lahti M et al., 2017 | Depressive Symptoms (before and during pregnancy) | Psychiatric Problems (1.5 to 5 years) |  | CE (95% CI) |  |  |  |
|  |  |  | Internalising Problems | CE 0.28(0.24;0.32),<0.001 |  |  |  |
|  |  |  | Externalising Problems | CE 0.26(0.22;0.30),<0.001 |  |  |  |
|  |  |  | Anxious/Depressed | CE 0.56(0.44;0.68),<0.001 |  |  |  |
|  |  |  |  |  |  |  |  |
| Giessen JV et al., 2019 | Inflammatory bowel disease(IBM) (before and during pregnancy) | Health-related quality of life (birth to 5 years) | Preschool Children Quality of Life Questionnaire(TAPQOL) | With IBD ,n(min-max) | Without IBD, n(min-max) |  |  |
|  |  |  | Total score | 90(86.5;93.3) | 89.1(84.4;92.3),>0.05 |  |  |
|  |  |  |  |  |  |  |  |
| Lyngsoe BK et al., 2019 | Depression(during pregnancy) | Health service use (birth to 2 years) |  | Recent depression, IRR(95%CI) | Previous depression IRR(95%CI) | Past depression IRR(95%CI) |  |
|  |  |  | General practice visits | IRR 1.16(1.15;1.17) | IRR 1.13(1.13;1.14) | IRR 1.15(1.14;1.16) |  |
|  |  |  |  |  |  |  |  |
| Simas TAM et al., 2019 | Postpartum depression (12 months after birth) | Health service use and costs (birth to 2 years) |  | PPD- Mean(SD) | Non-PPD Mean(SD) | P |  |
|  |  |  | Admissions(visits) | 0.9(0.7) | 0.8(0.7) | 0.004 |  |
|  |  |  | Outpatients | 1.0(1.5) | 0.8(1.4) | <.001 |  |
|  |  |  | Physician visits | 20.8(10.2) | 19.0(9.2) | <.001 |  |
|  |  |  | Pharmacy claims | 9.9(10.8) | 8.3(9.6) | <.001 |  |
|  |  |  |  |  |  |  |  |
|  |  |  | Inpatient admissions (cost per sector) | $23,856 | $21,388 |  |  |
|  |  |  | Outpatient service visits | $8,991 | $7,779 |  |  |
|  |  |  | Physician visits | $2,448 | $2,227 |  |  |
|  |  |  | Well-child office visits |  |  |  |  |
|  |  |  | Pharmacy claims | $716 | $559 |  |  |
|  |  |  |  |  |  |  |  |
| Hope H et al., 2021 | Mental illness (after birth) | Health service use and costs(birth to 17 years) |  | <1 year, RR(95%CI) | 1-4 years, RR(95%CI) | 5-9 years, RR(95%CI) |  |
|  |  |  | Any MMI | RR 1.21(1.20;1.22) | RR 1.19(1.18;1.20) | RR 1.28(1.26;1.30) |  |
|  |  |  | Common MMI | RR 1.21(1.20;1.22) | RR 1.19(1.18;1.20) | RR 1.28(1.26;1.30) |  |
|  |  |  | Serious MMI | RR 1.12(1.05;1.20) | RR 1.22(1.16;1.29) | RR 1.49(1.38;1.61) |  |
|  |  |  | Addiction disorders | RR 1.20(1.13;1.27) | RR 1.12(1.07;1.18) | RR 1.19(1.15;1.33) |  |
|  |  |  |  |  |  |  |  |
|  |  |  | Admissions(visits) | RR 1.52(1.48;1.57) | RR 1.39(1.33;1.44) | RR 1.35(1.27;1.43) |  |
|  |  |  | Outpatients | RR 1.22(1.19;1.25) | RR 1.34(1.31;1.37) | RR 1.36(1.33;1.39) |  |
|  |  |  | Primary care | RR 1.14(1.14;1.15) | RR 1.15(1.15;1.16) | RR 1.26(1.24;1.27) |  |
|  |  |  | Prescriptions | RR 1.29(1.27;1.30) | RR 1.19(1.17;1.20) | RR 1.27(1.24;1.29) |  |
|  |  |  | Emergency | RR 1.14(1.14;1.15) | RR 1.15(1.14;1.15) | RR 1.26(1.24;1.27) |  |
|  |  |  |  |  |  |  |  |
|  |  |  | Admissions(cost per child) | £1,448 | £507 | £233 |  |
|  |  |  | Outpatients | £1,161 | £387 | £383 |  |
|  |  |  | Primary care | £292 | £124 | £67 |  |
|  |  |  | Prescriptions | £82 | £44 | £42 |  |
|  |  |  | Emergency | £93 | £66 | £38 |  |
